# Recurrence of Preeclampsia in Northern Tanzania: A Registry-Based Cohort Study

**DOI:** 10.1371/journal.pone.0079116

**Published:** 2013-11-01

**Authors:** Michael J. Mahande, Anne K. Daltveit, Blandina T. Mmbaga, Gileard Masenga, Joseph Obure, Rachel Manongi, Rolv T. Lie

**Affiliations:** 1 Kilimanjaro Christian Medical University College, Moshi, Tanzania; 2 Department of Global Public Health and Primary Care, University of Bergen, Bergen, Norway; 3 Centre for International Health, University of Bergen, Bergen, Norway; 4 Norwegian Institute of Public Health, Oslo, Norway; 5 Department of Obstetrics and Gynaecology, Kilimanjaro Christian Medical Centre, Moshi, Tanzania; 6 Department of Pediatrics, Kilimanjaro Christian Medical Centre, Moshi, Tanzania; Yale School of Public Health, United States of America

## Abstract

**Objective:**

Preeclampsia occurs in about 4 per cent of pregnancies worldwide, and may have particularly serious consequences for women in Africa. Studies in western countries have shown that women with preeclampsia in one pregnancy have a substantially increased risk of preeclampsia in subsequent pregnancies. We estimate the recurrence risks of preeclampsia in data from Northern Tanzania.

**Methods:**

A prospective cohort study was designed using 19,811 women who delivered singleton infants at a hospital in Northern Tanzania between 2000and2008. A total of 3,909 women were recorded with subsequent deliveries in the hospital with follow up through 2010. Adjusted recurrence risks of preeclampsia were computed using regression models.

**Results:**

The absolute recurrence risk of preeclampsia was25%, which was 9.2-fold (95% CI: 6.4 - 13.2) compared with the risk for women without prior preeclampsia. When there were signs that the preeclampsia in a previous pregnancy had been serious either because the baby was delivered preterm or had died in the perinatal period, the recurrence risk of preeclampsia was even higher. Women who had preeclampsia had increased risk of a series of adverse pregnancy outcomes in future pregnancies. These include perinatal death (RR= 4.3), a baby with low birth weight (RR= 3.5), or a preterm birth (RR= 2.5). These risks were only partly explained by recurrence of preeclampsia.

**Conclusions:**

Preeclampsia in one pregnancy is a strong predictor for preeclampsia and other adverse pregnancy outcomes in subsequent pregnancies in Tanzania. Women with previous preeclampsia may benefit from close follow-up during their pregnancies.

## Introduction

Preeclampsia is associated with increased risk of maternal and perinatal morbidity and mortality, as well as long-term complications [1]. Preeclampsia is a major contributor to death and disability among women of reproductive age in many low income countries [2]. A review of preeclampsia studies in developing countries found prevalence ranging from 1.8% to 16.7% [3]. The highest figures are approximately 3-fold of the global prevalence of 4% [4].Preeclampsia remains a major public health problem in sub Saharan Africa.

Several risk factors for preeclampsia have been well documented [5,6]. These include first pregnancy, a history of preeclampsia, high maternal age, long inter-pregnancy interval, multiple pregnancy, gestational diabetes, chronic hypertension, family history of preeclampsia and history of preterm delivery. High body weight has been documented to be a risk factor also in Africa [7].

High recurrence risk of preeclampsia has been reported from high-income countries[8,9].Recurrent preeclampsia is also associated with adverse pregnancy outcomes in the subsequent pregnancies such as preterm birth, low birth weight, perinatal death and chronic hypertension [10,11]. 

There are limited data about recurrence of preeclampsia in low-income countries including Tanzania. Some studies in Africa have reported recurrence risks of hypertension [12,13]. However, these studies were based on cross-sectional data and had limited sample size.

Information on recurrence risk may help clinicians in decision making, for example in counselling women with previous history of preeclampsia who desire to have next pregnancy and in the clinical follow-up of these pregnancies. This study used prospective data from a hospital based registry in Tanzania to estimate the recurrence risk of preeclampsia. 

## Materials and Methods

A prospective cohort study was designed using maternally linked data from Kilimanjaro Christian Medical Centre (KCMC) medical birth registry. A unique maternal hospital identification number was used to link records of subsequent deliveries of the same mother.

KCMC is one of the four zone referral hospitals in Tanzania, located in Moshi urban district, Kilimanjaro region in the Northern Tanzania. The centre receives deliveries from the nearby communities within the region and referred cases from other regions (i.e. from more distant areas). We excluded women who were referred from more distant areas for various medical reasons, since medical problems could be overrepresented in our data among women from more distant areas. We also excluded women with multiple gestations. The study population was women from the natural catchment area of KCMC with singleton deliveries.

The medical registry collects information from all women who deliver at the department of obstetrics and gynecology within 24 hours after delivery or as soon as mothers have recovered in case of complicated deliveries. Trained nurse midwives carried out daily interviews using a standardized questionnaire to collect the registry data. In addition, mothers admitted were asked to provide their antenatal (ANC) cards from which relevant data were abstracted. Furthermore, data were abstracted from patient case notes housed in the medical records.The details of data collection methods have been described elsewhere [14]. In summary, data captured in the standardized questionnaire include parents’ socio-demographic characteristics, maternal health before and during present pregnancy, complications during labour and delivery, and information from the interview regarding the mother’s previous pregnancies. In addition, information on the baby such as sex, date and time of delivery, birth weight, gestational age, presentation, length and head circumference, plurality, mode of delivery, abnormal conditions (birth defects, injuries or other diseases), Apgar score, and child status in four categories: 1) live born 2) live born transferred to NCU 3) neonatal death in labour ward, 4) stillborn were recorded.

**Figure 1:**
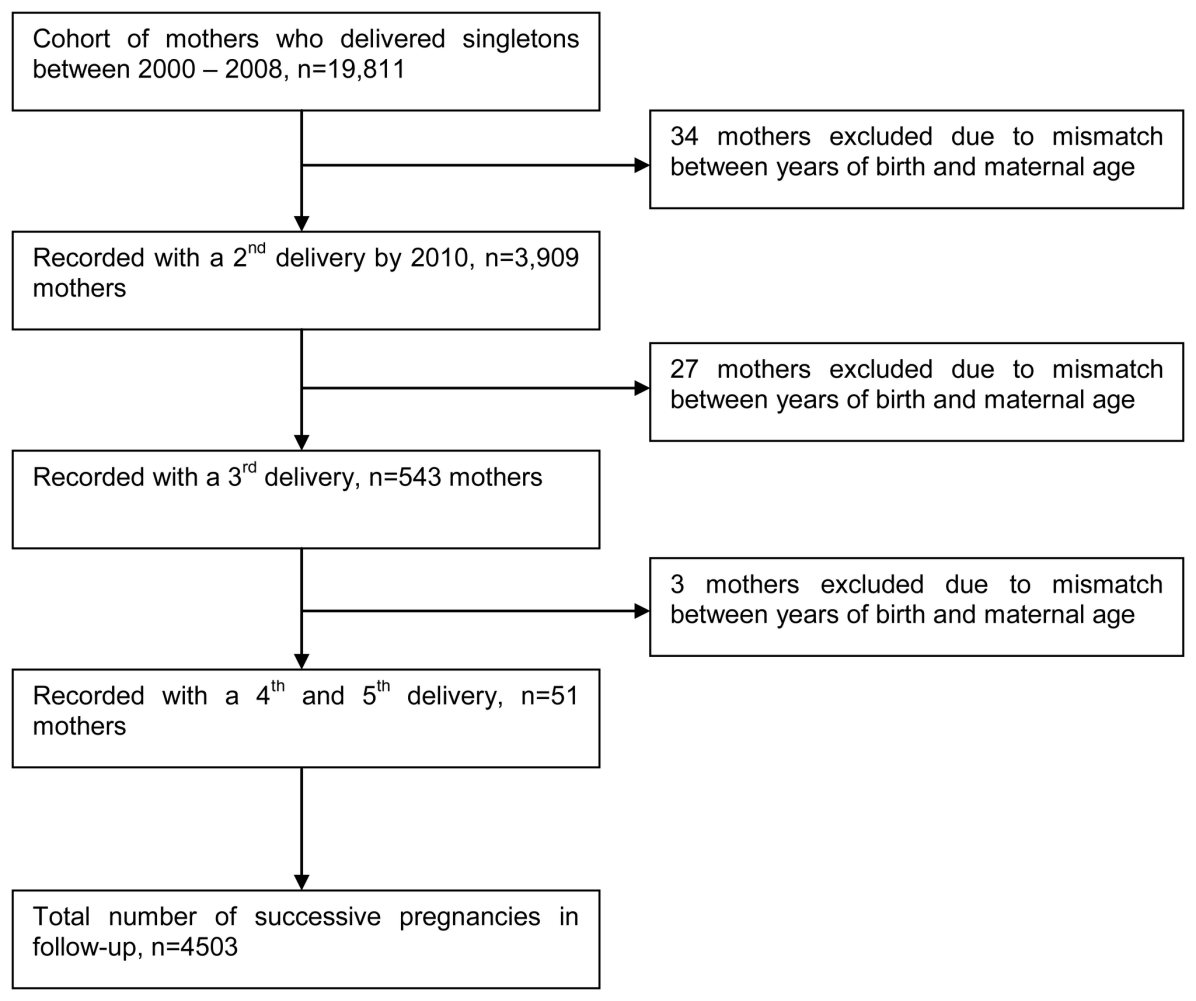
Schematic presentation of the cohort follow-up. Data from the Kilimanjaro Christian Medical Centre (KCMC) birth registry.

We constructed our cohort using 19,811 (736; 3.7% with preeclampsia and 19,075 without preeclampsia) women who were recorded for the first time with a singleton delivery at KCMC in the period 2000–2008.The women were then followed for any subsequent birth sin the hospital up to 2010 using the unique maternal hospital number. The median follow-up window period was 6.5 years. We excluded women who were referred from rural areas for various medical reasons and those with multiple gestations. Our final study sample was 3,909 (19.7%) of women whom were recorded with at least one or more birth during the follow-up period (Figure **1**). These women had a total of 4,503 additional pregnancies in the follow-up period. We studied recurrence of preeclampsia from the first recorded pregnancy to any of the subsequent pregnancies. 

In order to ensure that there was a true linkage between pregnancies of the same mother, we used various validation criteria such as; 1) matching the year of birth in the linked data with the same year recorded in the reproductive history data based on maternal recall and interview after each birth; 2) birth interval calculated from birth dates were cross-checked against the recorded change in maternal age for 2 consecutive deliveries, and a discrepancy of more than 2 years led to exclusion from the data.

Our ability to capture subsequent births at the hospital may have been incomplete. By using reproductive history interviews conducted at each delivery we were able to calculate the expected proportion of women with a subsequent birth within the follow-up time. Assuming symmetry of the distribution of births within the period 2000 to 2010, we constructed a “backwards cohort” by identifying the last birth of 21,086 mothers in the period 2002-2010. A total of 7,191 women (34.1%) reported a prior birth in the period 2000-2010. Comparing this estimate with the 19.7% who actually were observed with a subsequent birth we estimated that the completeness of our follow-up was 58% (19.7/34.1).

The main outcome variable was relative risk and recurrence risk of preeclampsia in subsequent pregnancies. Preeclampsia was in our study defined as increased blood pressure to a level of 140 mmHg systolic blood pressure or more and diastolic blood pressure 90 mmHg or above) recorded after 20 weeks’ of gestationage combined with proteinuria (≥300mg in a 24 hours urine collection) [15].Recurrence of preeclampsia was defined as repetition of a preeclampsia diagnosis in a subsequent pregnancy.

Data were analyzed using SPSS package version 18.0 (SPSS Inc., in Chicago, Illinois, USA) and Stata version 12.0. Student’s t-test was used for comparison of continuous variables, and comparison of proportions was performed by chi-square (χ^2^).Log-binomial regression model was used to estimate the relative risk and recurrence risk for preeclampsia with95% confidence intervals (CIs).A p-value of less than 5% was considered statistically significant. The recurrence of preeclampsia was estimated from the first to any of the subsequent pregnancies. We used mothers as the primary unit for our analysis and conducted a clustered analysis technique with robust estimation of variances to account for the correlation between repeated observations of the same woman in the follow-up period.

### Ethics Statement

This study was approved by the Kilimanjaro Christian Medical University college ethics committee and by the National Institute of Medical Research of Tanzania. For practical reasons, the midwives give oral information individually to each mother and then asked if is willing to participate. Participation and further interviewing was then based on oral consent. Following consent, the mother could still choose not to reply to single question. In order to document the consent process, the birth registry has an instruction manual for registration of deliveries in the registry. This manual contains the standard operating procedures on how to conduct the interview and also has a description of the basic rules including ethical guidelines such as consent process. The consent procedure was approved by the ethics committee as it was part of the study protocol. Approvals were given by the local IRB at the hospital and by the Tanzanian Ministry of Health. Since the project received Norwegian funding, the protocol was also submitted to a Norwegian IRB which stated that they did not need to approve the project. 

## Results

Our study included a total of 3,909 women who were recorded in the registry with at least two singleton births during the study period. Among these women, 137 (3.5%) had preeclampsia in their first pregnancy. Maternal and fetal characteristics of the first pregnancy are shown in Table **1**.Women with preeclampsia in their first recorded pregnancy were older (P=0.001) and had shorter gestation age at delivery (P<0.001) compared with women who did not have preeclampsia. Furthermore, women with preeclampsiahad significantly higher rates of chronic hypertension, perinatal death, induced labour, preterm birth and were more likely deliver babies with low birth weight. The 3,909 women in our cohort had a total of 4,503 subsequent pregnancies recorded in our data that were used to study recurrence.

**Table 1:** Socio-demographic and obstetric factors of the 3,909 women in the cohort.

	Outcome in the 1^st^recorded pregnancy		
Maternal characteristics in 1^st^recorded pregnancy	No preeclampsia n (%)	Preeclampsia n (%)	P- value*
Total	3,772(96.5)	137 (3.5)	
Education level:			0.04
≤12 years	2,514	75 (2.8)	
>12 years	1,258	62 (4.7)	
Pregnancy Body Mass Index (BMI)^∞^			0.09
Underweight (<18.5)	499	22 (4.2)	
Normal (18.5-24.9)	198	5 (2.5)	
Overweight (25-29.9)	276	7 (2.5)	
Obese (≥30)	202	14 (6.5)	
Number of ANC visits:			0.24
<5	2,216	87 (3.8)	
≥5	1,556	50 (3.1)	
Gestation hypertension			<0.001
Yes	14	4 (22.0)	
No	3,758	133 (3.4)	
Chronic hypertension			<0.001
Yes	36	11 (23.4)	
No	3,736	126 (3.3)	
Diabetes			
Yes	5	2 (28.6)	<0.001
No	3,767	135 (3.5)	
Induced labour			0.003
Yes	1,447	70 (4.6)	
No	2,325	678 (2.8)	
Caesarian section			0.24
Yes	1,199	50 (4.0)	
No	2,573	87 (3.3)	
Preterm birth^§^			<0.001
Yes	462	47 (9.2)	
No	3,019	80 (2.6)	
Low birth weight	518	56 (9.8)	<0.001
Yes	3,254	81 (2.5)	
No			
Perinatal death			<0.001
Yes	245	26 (9.6)	
No	3,527	111 (3.1)	
Maternal age: mean (SD)	25.9 (4.9)^†^	27.4 (4.9)^†^	0.001
Gestational age at delivery	38.9 (2.7) ^†^	37.0 (3.3)	<0.001

* Chi-square tests for heterogeneity except of a t-test for mean maternal age

† SD – Standard Deviation

§ Does not add to total because 301 (7.7%) missed gestational age

∞Does not add to total because of missing BMI variables (weight or height)

First, we studied how the risk of preeclampsia in a future pregnancy was determined by maternal and fetal conditions of the first recorded pregnancy (Table **2**). The absolute recurrence risk of preeclampsia was 24.6%, which amounted to a 9.2-fold relative risk (95% CI: 6.4 - 13.2). In a sub analysis, we stratified the recurrence risk for preeclampsia by order of subsequent pregnancy. The recurrence risk of preeclampsia in the second pregnancy was 10.3-fold (95% CI: 7.3-14.8), for third, fourth or fifth pregnancy combined, the risk was 5.0-fold (95% CI: 2.1-12.0). These relative risks were, however, not significantly different (p=0.10).

**Table 2:** Risk of preeclampsia by maternal characteristics in first pregnancy.

Characteristic of	Preeclampsia in subsequent pregnancy			
First pregnancy*	At risk	n (%)	RR (95 % CI)^†^	P-value
Preeclampsia				
Yes	171	42 (24.6)	9.2 (6.4 - 13.2)	<0.001
No	4,332	103 (2.4)	Reference	
Chronic hypertension				
Yes	63	18 (28.6)	8.9 (5.7 - 13.8)	<0.001
No	4,440	127 (2.9)	Reference	
Gestational hypertension				
Yes	25	9(36.0)	9.8 (4.9 - 19.1)	<0.001
No	4,478	136(3.0)	Reference	
Diabetes Mellitus				
Yes	8	2 (25.0)	8.4(2.7 - 26.3)	<0.001
No	4,495	143 (3.2)	Reference	
Preterm birth^§^				
Yes	610	45 (7.4)	3.1 (2.1 - 4.7)	<0.001
No	3,544	87 (2.5)	Reference	
Low birth weight (<2500g)				
Yes	689	51 (7.4)	3.1 (2.1 - 4.5)	<0.001
No	3,814	93 (2.4)	Reference	
Perinatal death				
Yes	341	36(10.6)	3.9 (2.7 - 5.9)	<0.001
No	4,162	109 (2.6)	Reference	

*Adjusted for maternal age and maternal education in Log-binomial model accounting for correlation between successive deliveries of the same mother.

^†^ RR=Adjusted Relative Risk, CI= Confidence Interval

^§^ Does not add to total because 349 (7.8%) missed gestational age

Another factor that may modify the recurrence risk is the inter-pregnancy interval, which also will be higher for recurrence to 3^rd^, 4^th^or 5^th^ pregnancy. The recurrence risk for a new pregnancy that started within the next four years was 10.0-fold (95% CI: 6.4-15.7). This was not significantly different from an estimated 8.3-fold risk (95% CI: 5.0-13.6) for a new pregnancy that started after more than four years.

Several other maternal conditions in the first pregnancy showed associations with increased risk of preeclampsia in a subsequent pregnancy that was similar to the recurrence risk. These included chronic hypertension (RR= 8.9; 95% CI: 5.7 -13.8), gestational hypertension (RR= 9.8; 95% CI: 4.9 -19.1) and diabetes mellitus (RR= 8.4; 95% CI: 2.7 - 26.3).

Fetal outcomes of first pregnancy were also associated with the risk of preeclampsia in a subsequent pregnancy. A preterm birth increased the risk 3.1-fold (95% CI: 2.1 - 4.7), low birth weight 3.1-fold (95% CI: 2.1 - 4.5) and perinatal death 3.9-fold (95% CI: 2.9 - 5.9).

**Figure 2:**
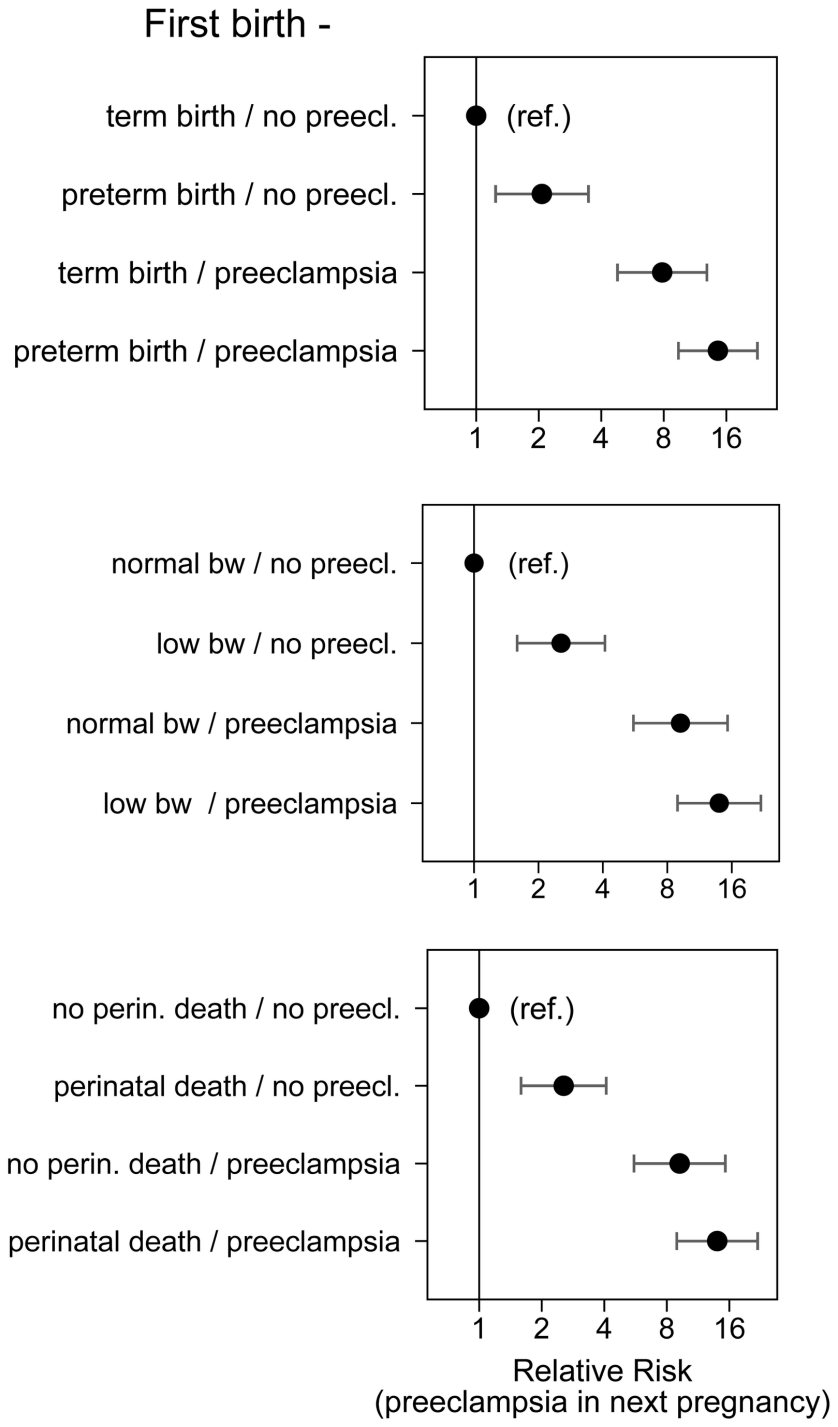
Conditions of the first pregnancy (preeclampsia in combination with preterm birth, low birth weight or perinatal death) and the risk of preeclampsia in the next pregnancy.

These risks depended, however, on the presence of preeclampsia in the first pregnancy. Figure **2** shows the risks of preeclampsia in a subsequent pregnancy by whether there was preterm birth or preeclampsia or both in the first pregnancy. The highest risk was seen when a woman had a first pregnancy with preeclampsia that was delivered preterm, perhaps because serious preeclampsia has higher recurrence risk (absolute recurrence risk of 35.5%). Slightly lower risk was seen after a first pregnancy with preeclampsia that was delivered at term. A preterm birth without any recorded preeclampsia still increased the risk of preeclampsia in a future pregnancy. Similar patterns of risk were seen when preeclampsia in first pregnancy was combined with low birth weight or perinatal death. The effects of low birth weight were similar when we restricted to term births.

**Figure 3:**
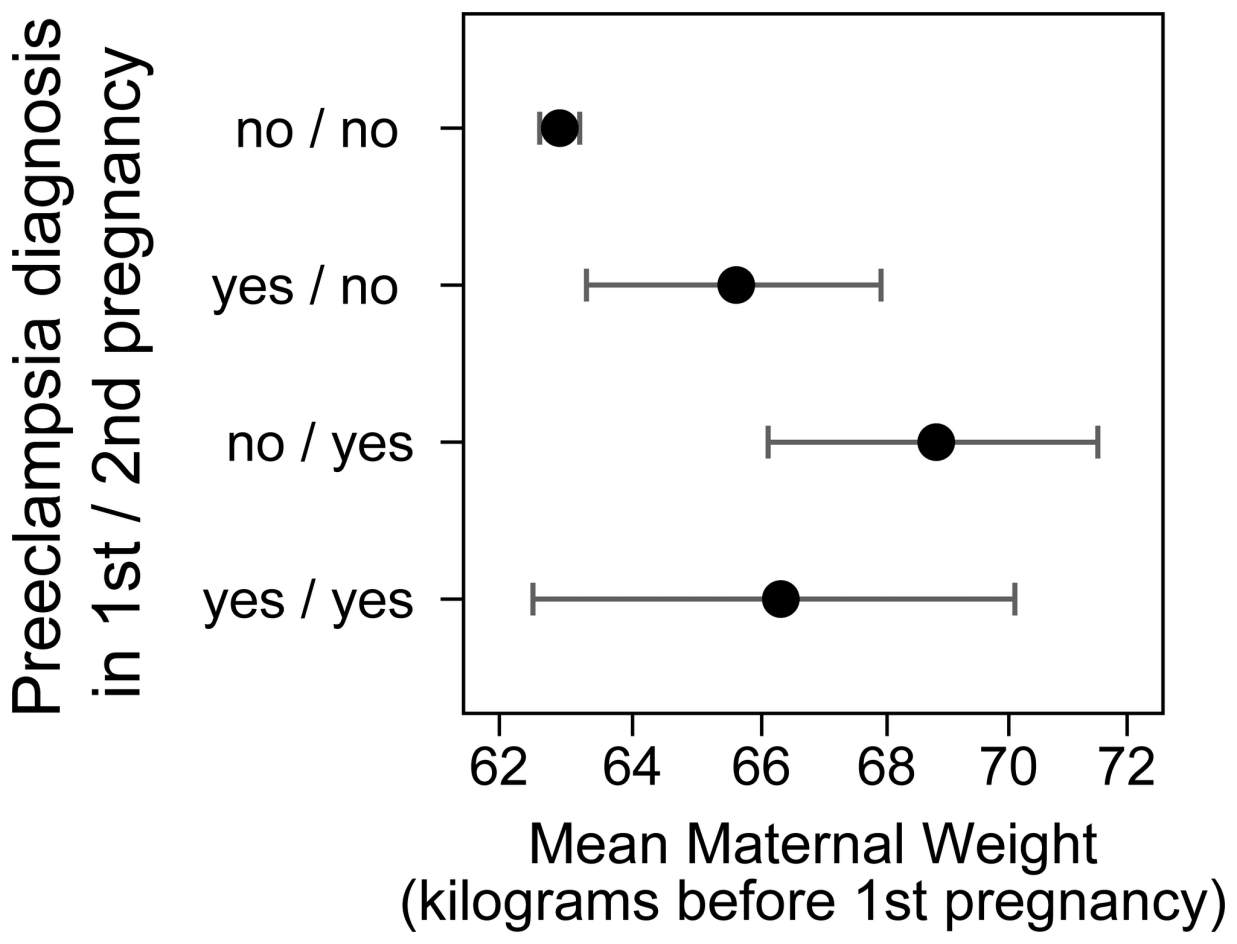
Mean of mother’s body weight before the first pregnancy for women with two recorded pregnancies depending on whether each pregnancy was affected by preeclampsia.

High body weight is a known risk factor for preeclampsia. We explored whether body weight before first pregnancy was associated with risk of recurrence of preeclampsia. Women who went on to have two pregnancies, both affected by preeclampsia, had an average weight before the first pregnancy of 66.3 kilograms. This was not different from 65.6 kilograms for women who had preeclampsia only in the first of two pregnancies (Figure **3**). There was also little difference for these two groups in weight before the second pregnancy. Although higher weight is associated with preeclampsia, future recurrent preeclampsia could not be predicted by high body weight of a woman in our data.

We then went on to study the effect of preeclampsia in the first pregnancy on the risks for babies of future pregnancies (Table **3**). For women who had preeclampsia in their first pregnancy, the total risk of preterm birth, low birth weight or perinatal death in a subsequent pregnancy increased 2.5-fold, 3.5-fold and 4.3-fold, respectively. As seen from the previous analysis, this could be partly explained by recurrence of preeclampsia. When we adjusted for a diagnosis of preeclampsia in the subsequent pregnancy (in addition to the other adjustments) we expect to remove the contribution of recurrent preeclampsia to these risks. The new estimates were lower, but still two- to three-fold with confidence intervals excluding one. We denoted these risks “direct risks” in the table to indicate that these associations do not appear to work through recurrence of a diagnosis of preeclampsia.

**Table 3:** Preeclampsia and risk of adverse fetal outcomes in a future pregnancy.

			Risk in future pregnancy			
Outcome in future pregnancy	Preeclampsia in 1^st^ pregnancy	At risk		n (%)	RR(95 % CI)* “Total risk”	RR (95% CI) ^†^ “Direct risk”
Preterm birth^§^	Yes	162		28 (17.3)	2.5 (1.7-3.6)	1.8 (1.2-2.7)
	No	4,023		299 (7.4)	Ref.	Ref.
Low birth weight	Yes	171		24 (14.0)	3.5(2.2-5.4)	1.8 (1.1-3.1)
	No	4,329		174 (4.0)	Ref.	Ref.
Perinatal death	Yes	171		22 (12.9)	4.3(2.7-6.8)	3.1(1.8-5.3)
	No	4,332		125 (2.9)	Ref.	Ref.

* “Total” relative risk adjusted for maternal age, maternal education, and chronic hypertension in a future pregnancy using a log-binomial model and accounting for correlation between successive deliveries of the same mother.

† “Direct” relative risk adjusted also for preeclampsia in a future pregnancy to remove the contribution working indirectly through recurrence of preeclampsia

§ Lower numbers because of missing gestational age information

## Discussion

Our data show that preeclampsia is not only a serious complication of a particular pregnancy in Tanzania, but also a strong predictor for preeclampsia and other adverse outcomes in future pregnancies. Women with preeclampsia in a previous pregnancy had a 9-fold increased risk of preeclampsia. The absolute recurrence risk was as high as 25%.In addition, when there were signs that preeclampsia in the previous pregnancy had serious consequences; the risk of preeclampsia in future pregnancies was even higher.

Cross-sectional studies in Africa have reported recurrence risks of preeclampsia and hypertension, ranging from15.8% to 36% [13,16]. The estimated recurrence risk of preeclampsia in our prospective study falls within this range. The absolute recurrence risk of preeclampsia observed in our study was higher than estimates from studies in western countries [11,17]. For example, studies in Scandinavian countries have found recurrence risks in the range of 13% to 15% [9,18-20].Other studies in western countries have reported a recurrence of preeclampsia of 14% [21,22]. A recent study in Israel reported the recurrence risk of preeclampsia as low as 6% [15].Our estimate from Tanzania was, however, not very different from 28% reported in USA [10]. Possible explanations for these differences in recurrence may be the differences in prevalence of risk factors for preeclampsia between the studied populations such as chronic hypertension and diabetes or differences in diagnosis. Lower absolute risk of recurrence could also be attributed to heightened antepartum surveillance system for mothers with previous preeclampsia who are considered as high risk group by health care providers. It is also possible that poor management of preeclampsia in one pregnancy increased the risk of preeclampsia in the next pregnancy in some countries.

We show in our data that the recurrence risk varies by assumed severity of preeclampsia and by presence of chronic conditions like hypertension and diabetes. Similarly, Mamomed and colleagues [12] reported that history of chronic hypertension was associated with eleven-fold increased risk of preeclampsia. Other studies also report high recurrence risk after a preeclamptic pregnancy that was delivered preterm [11,15,20]. One possible reason for discrepancy between our estimated recurrence risk and those of some others studies may be differences in the proportion of serious preeclampsia cases included in the studies. Our hospital data may contain more women with very serious preeclampsia than in the general population.

We found that a history of preeclampsia was associated not only with future risk of preeclampsia but also withadverse outcomes for future babies such as preterm birth, perinatal death and low birth weight. Our finding is in agreement with previous studies [11,15,20].We also show that this increased risk is not only due to the effects of recurrent preeclampsia.

Women should receive counselling after having had preeclampsia about the future risks. There is also apotential to use the relatively frequent antenatal visits in this population to detect the development of preeclampsia in women who had preeclampsia in a previous pregnancy and who would benefit from closer clinical monitoring. Measures to prevent recurrent preeclampsia have been proposed [8,23], but the utility of each of these needs to be studied in an African context.Our study also suggests benefits of closer clinical follow up for the babies of future pregnancies.

Our study had a number of limitations that need to be considered. First, the possibility of selection bias is an inherent problem in hospital-based studies as compared to population-based studies. Our study excluded all women who were referred for various medical conditions. We found no effect of socio-economic status on recurrence risks. Still, our study may not be representative of the whole population in the area.

Second, a loss to follow-up estimate of 42%indicates that a high proportion of women were not recorded with their subsequent pregnancies. These women may have had different risk characteristics from those who were followed up. The proportion of women who showed up for a future birth were, however, not very different for women with previous preeclampsia (19%) and women with no previous preeclampsia (20%). We also repeated our analysis by excluding all women who were referred for various medical reasons in their second pregnancy to minimize selection bias due to referral of future births. The relative risk of recurrence of preeclampsia remained unaffected, (9.2 vs. 8.7). This gave us some degree of assurance that the association is not much affected by bias due to selection in the follow-up. 

Third, our ability to identify future births of the same woman by record linkage may be imperfect. We excluded women who did not meet several matching criteria. Errors in hospital numbers and failed linkages may be one source of loss of follow-up in the present study. Such errors should, however, be random, and not affect our results. 

Our study also had several strengths. To our knowledge, this is the largest prospective cohort to study recurrence risk of preeclampsia in Tanzania, and perhaps even in sub Saharan Africa. Our study provided estimates that seemed to be consistent with the literature. By linking maternal and sibling records, we were able to calculate recurrence risks of preeclampsia, which would not be reliable with cross sectional data. Our study was not affected by recall bias. 

The KCMC birth registry used a standardized data collection mechanism with the same staff over more than a decade. Detailed information regarding reproductive outcomes of successive pregnancies of each woman was captured by linking birth records using a unique maternal identification number and other specific matching criteria.

In conclusion, preeclampsia in one pregnancy was a strong risk factor for preeclampsia in future pregnancies in Tanzania. The risk increased by severity of the condition. Preeclampsia also increased the risk for the babies of future pregnancies. Our study suggests that improved clinical counselling and management of pregnant women with a history of preeclampsia may benefit women in Tanzania and their babies. 
